# Sliding and Fretting Wear Behavior of Biomedical Ultrafine-Grained TiNbZrTaFe/Si Alloys in Simulated Physiological Solution

**DOI:** 10.3390/ma17040787

**Published:** 2024-02-06

**Authors:** Yuhua Li, Qian Zhang, Yuxin He, Rong Zhao, Jinghui Chu, Libin Niu, Juxin Qu

**Affiliations:** 1 College of Mechanical Engineering, Xi’an University of Science and Technology, Xi’an 710054, China; 2 National Engineering Research Center of Near-Net-Shape Forming for Metallic Materials, South China University of Technology, Guangzhou 510640, China

**Keywords:** biomaterials, titanium alloys, wear resistance, sliding and fretting, simulated physiological solution

## Abstract

This work investigated the wear behavior of ultrafine-grained Ti_65_Nb_23.33_Zr_5_Ta_1.67_Fe_5_ (at.%, TNZTF) and Ti_65_Nb_23.33_Zr_5_Ta_1.67_Si_5_ (at.%, TNZTS) alloys fabricated by high-energy ball milling and spark plasma sintering. Wear tests were conducted in a simulated physiological solution under both reciprocating sliding and fretting wear conditions with different loads, frequencies, and stroke lengths. The microstructures, mechanical properties, and anti-wear properties of the investigated alloys were characterized. The results showed that the TNZTF and TNZTS alloys had much less wear volume than the commonly used Ti-6Al-4V (TC4) alloy and commercially pure titanium (CP-Ti). The TNZTF and TNZTS alloys exhibited much more smooth wear surfaces and shallower wear scars compared with TC4 and CP-Ti. The investigated alloys exhibited different wear mechanisms under the reciprocating sliding wear conditions, while they were similar under the fretting wear conditions. Compared with TC4 and CP-Ti, the fabricated TNZTF and TNZTS alloys showed a substantially higher wear resistance, owing to their ultrafine-grained microstructure and superior hardness. Additionally, the addition of Nb and Zr further enhanced the wear resistance by forming a protective Nb_2_O_5_ and ZrO_2_ oxide film. This work provides guidance for designing new biomedical titanium alloys with excellent wear resistance.

## 1. Introduction

Titanium alloys are potential materials for biomedical applications such as joint prostheses, owing to low elastic modulus, high specific strength, and excellent biocompatibility [1,2,3,4,5,6,7,8,9]. Until recently, commercially pure titanium (CP-Ti) and Ti-6Al-4V (TC4) alloy have been preferred in biomedical applications such as dental implants, joint replacement parts, and bone fixation materials. However, interest in these materials has gradually decreased due to their high elastic modulus and the release of Al and V from TC4 into the human body [8,10]. To improve the biocompatibility and solve the present problems, β-Ti alloys have been developed by adding non-toxic elements such as Nb, Zr, Mo, and Ta [1,2,3,5,6,7,8,11].

The disadvantage of β-Ti alloys may be the insufficient wear-resistant properties. Çomaklı et al. [12] reported that although Ti45Nb alloys possess excellent mechanical and biocompatibility properties, Ti45Nb exhibits insufficient surface properties for biomedical applications. Its low tribological performance leads to wear debris, which can result in a number of health problems such as inflammation, toxicity, and bone loss. Excessive wear of artificial joints in the body has been a major problem affecting joint replacement life [13,14]. The abrasion of the artificial joint may cause the immune reaction of tissues around the body and even toxicity, leading to bone absorption and loosening [15].

To avoid this phenomenon, many techniques have been developed to improve the wear resistance of implants [16,17,18]. Surface modification of titanium alloys can be achieved through conventional and non-conventional technologies, including anodisation [19], laser cladding technology [20], plasma spraying [21], physical vapor deposition (PVD) and chemical vapor deposition (CVD) technologies [22], nitriding [23], micro-arc oxidation (MAO) [24], and high-velocity oxygen fuel spray (HVOF) [25]. However, such surface treatments sometimes induce some harmful effects on other properties except for wear resistance [26]. These coatings have been reported to be problematic in attachment to the base material, increasing wear and chipping [27]. Nanostructured titanium alloy surfaces have potential to be incorporated on orthopedic implants and confer to the devices antibacterial and osseointegration properties [28,29,30,31]. Moreover, nanostructured titanium alloy surfaces could improve wear resistance [32,33]. Some of the newly developed β-Ti alloys still lack enough wear resistance [7,8,16,18,34,35], and the biomedical application remains somewhat limited [8]. Developing ultrafine-grained and nanostructured titanium alloys has the benefit of improving wear resistance [32], being necessary for biomedical applications. A better understanding of the mechanisms involving the friction and wear of titanium alloys is required to improve tribological properties. Extensive studies have been made on the wear behaviors of CP-Ti, TC4, Ti-Nb-Ta-Zr, Ti-Nb-Zr, Ti-Zr-Hf-Nb-Fe, and Ti-Nb-Ga alloys in both dry and wet conditions [13,14,36,37,38,39,40]. Many reports so far have focused on the tribological behaviors of titanium alloys under reciprocating sliding or fretting wear conditions [41,42,43,44,45].

Knee and hip joint replacement implants involve a sliding contact between the femoral component and the tibial or acetabular component immersed in body fluids, thus making the metallic parts susceptible to friction and abrasion [46]. Micro-motions occur at points of fixation, leading to debris and ion release by fretting wear. Fretting is defined as the small-scale oscillatory movement between two contacting surfaces (typically less than 100 μm) that are typically not intended to move [47]. The metal surfaces of these junctions are subjected to sliding and fretting wear, which may lead to early failure. Therefore, both sliding and fretting wear conditions are necessary to be considered for biomedical applications [46].

To study the wear behavior of metallic biomaterials, ceramic materials are frequently selected as the friction pair, such as zirconia [48,49,50,51,52], alumina [39,41,45,46,53], and silicon nitride [36,38,43]. Zirconia presents the highest toughness among ceramic materials in addition to high hardness [54]. This type of ceramic materials is selected to limit severe adhesive wear, which typically occurs during metal–metal contact [34,37,55]. Moreover, the ceramic materials have higher mechanical properties than the titanium alloys, and therefore wear is exclusively expected on the metal specimens.

As such, in this work, sliding and fretting wear studies were performed on the newly developed Ti_65_Nb_23.33_Zr_5_Ta_1.67_Fe_5_ (at.%, TNZTF) and Ti_65_Nb_23.33_Zr_5_Ta_1.67_Si_5_ (at.%, TNZTS) in the simulated physiological environment. By using zirconia balls as a counterpart, the sliding contact of the ball/acetabular cup and fretting contact of the femoral stem/ball were both simulated. The TNZTF and TNZTS alloys were fabricated by high-energy ball milling and spark plasma sintering. The anti-wear properties and wear mechanisms of the fabricated TNZTF and TNZTS alloys were investigated and compared with those of the TC4 alloy and CP-Ti samples because TC4 and CP-Ti are commonly used as orthopedic implant materials. Wear tests were performed in Hank’s balanced salt solution at the body temperature of about 37 ± 1 °C under reciprocating sliding and fretting wear conditions with different loads, frequencies, and stroke lengths.

## 2. Experimental Methods

### 2.1. Fabrication and Characteristics of the Bulk TNZTF and TNZTS Alloys

The ultrafine-grained TNZTF and TNZTS alloys were fabricated by spark plasma sintering and crystallizing metallic glass (MG) powder. Firstly, the TNZTF and TNZTS MG powders were prepared from respective elemental powders by mechanical alloying. The detailed synthesis procedures of the MG powders can be seen in our previous work [56,57]. Under the protection of Ar atmosphere, the as-prepared MG powders were sintered via a Dr. Sintering SPS-825 system (Sumitomo Coal Mining Co. Ltd., Tokyo, Japan). To obtain the ultrafine-grained structure, the sintering parameters were set as heating to 1233 K with a heating rate of 250 K/min, a sintering pressure of 50 MPa, and a holding time of 5 min. The sintered bulk alloys of Φ20 × 12 mm were obtained. As a control, the TC4 and CP-Ti samples with a diameter of Φ20 mm were provided via the Northwest Institute for Non-Ferrous Metal Research (Xi’an, China).

Phase constituents of the bulk alloys were characterized utilizing X-ray diffraction (XRD) (D/Max-2500pc, Rigaku Corporation, Tokyo, Japan) with Cu *Kα* radiation (λ = 0.15418 nm). The microstructures of the bulk alloys were observed using a scanning electron microscope (SEM) (Nova NanoSEM430, FEI, Hillsboro, OR, USA). Vickers hardness was measured via an HVS-1000 micro-hardness testing machine (Wolpert Wilson Instruments, Norwood, MA, USA) at a load of 2.94 N and holding time of 20 s. Mechanical properties of the bulk alloys of Φ3 × 6 mm were analyzed by uniaxial compression testing via an MTS Test Star 810 testing system at a strain rate of 5 × 10^−4^ s^−1^. The detailed phase constituents and mechanical properties of the bulk alloys are listed in Table 1.

### 2.2. Wear Tests

The anti-wear properties were analyzed via wear tests using the ball-on-disc UMT-3 Muti-Specimen Test System (Bruker, Billerica, MA, USA). The specimens of Φ10 × 6 mm were used as static disks. All wear tests were conducted in Hank’s balanced salt solution (pH = 7.3) at 37 ± 1 °C. The composition of the Hank’s balanced salt solution is listed in Table 2.

Before the wear tests, the specimens were progressively ground with emery papers up to 2000 grits and finely polished with diamond paste (0.1 μm) to achieve a surface roughness of about 0.02 μm. Zirconia balls (Beijing Zhongke Aobo Technology Co., Ltd., Beijing, China) of Φ12 mm in diameter were used as the moving counter bodies. The disks and balls were ultrasonically cleaned in acetone and ethanol for 10 min prior to the wear test, respectively.

**Figure 1:**
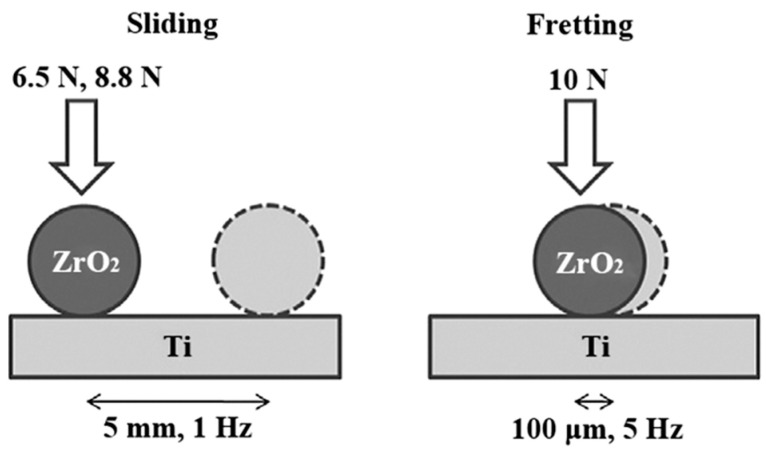
Schematic diagram of the wear test system.

A schematic diagram of the wear test system is shown in Figure 1. Sliding wear tests were carried out at respective loads of 6.5 N and 8.8 N, frequency of 1 Hz, and stroke length of 5 mm for 60 min. Under the sliding wear conditions, the initial maximum contact pressures were about 220 and 300 MPa. These two sliding wear conditions are referred to as 6.5 N-1 Hz-5 mm-60 min and 8.8 N-1 Hz-5 mm-60 min, respectively, hereafter. Fretting wear tests were performed at a load of 10 N, frequency of 5 Hz, and stroke of 100 μm for 60 min. Under the fretting wear condition, the initial maximum contact pressure was about 350 MPa. The fretting wear condition is referred to as 10 N-5 Hz-100 μm-60 min hereafter.

The samples after the wear tests were rinsed with ethanol before the various data acquisitions. The morphologies and anti-wear properties of the worn surface were analyzed using a MicroXAM-3D surface profiler (ADE Corporation, Westwood, MA, USA). The 3D optical micrographs were observed. The width and depth of wear scars were analyzed via scanning wear scar tracks. The wear volume was measured. The morphologies of the worn surface were also analyzed via SEM. The elemental compositions and chemical states of the worn surface were characterized using X-ray photoelectron spectroscopy (XPS) (Axis Ultra DLD, Kratos Corporation, Manchester, UK). Al Kα X-ray was performed as the source (hv = 1486.6 eV) at 15 kV and 10 mA. The pass energies of full-range spectrum scanning and narrow spectrum scanning were 160 eV and 40 eV, respectively. The vacuum of the analysis chamber was about 5 × 10^−9^ torr. The size of the spot was about 700 × 300 μm^2^. The C1s of 284.6 eV was used as the standard peak to calibrate the XPS spectra.

## 3. Results and Discussion

### 3.1. Phase and Microstructure Characterizations

**Figure 2:**
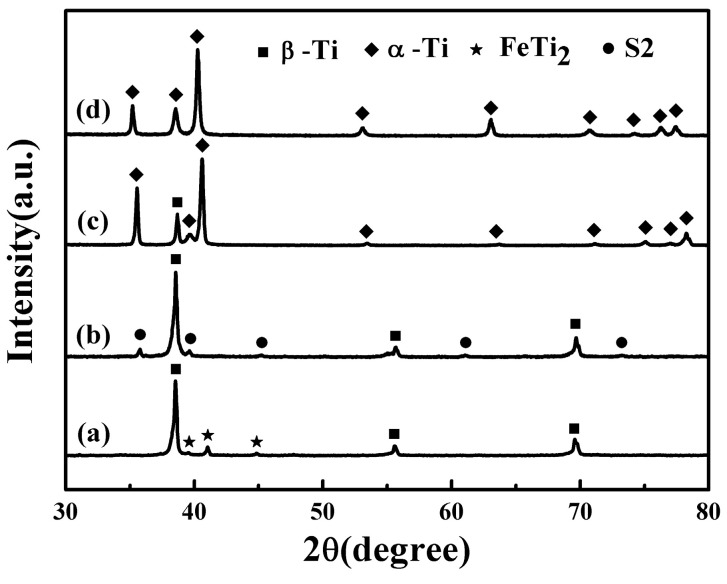
XRD patterns of the bulk samples: (a) TNZTF; (b) TNZTS; (c) TC4; (d) CP-Ti.

**Figure 3:**
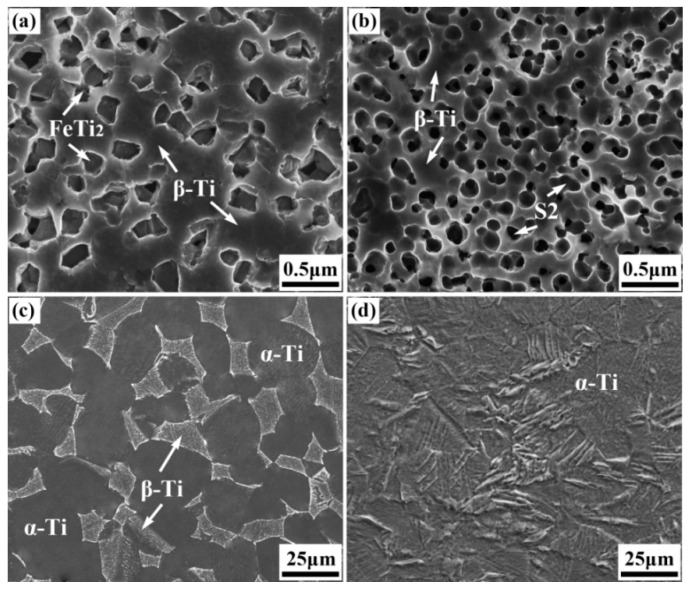
SEM microstructures of the bulk samples: (**a**) TNZTF; (**b**) TNZTS; (**c**) TC4; (**d**) CP-Ti.

Figure 2 and Figure 3 show the XRD patterns and SEM microstructures of the bulk samples before the wear test, respectively. The TNZTF alloy was mainly composed of a body-centered cubic (bcc) β-Ti matrix and intermetallic face-centered cubic (fcc) FeTi_2_ phase (Figure 2a and Figure 3a). The TNZTS alloy contained a hexagonal (Ti, Zr)_2_Si (S2) region surrounded by the bcc β-Ti matrix (Figure 2b and Figure 3b), which agreed well with the results in our previous work [56,57]. The TC4 alloy was composed of hexagonal close-packed (hcp) α-Ti phase and bcc β-Ti phase (Figure 2c and Figure 3c). The CP-Ti consisted of α-Ti phase (Figure 2d and Figure 3d). Obviously, the TC4 (Figure 3c) and CP-Ti (Figure 3d) samples had a much larger size of grain or phase region than the TNZTF and TNZTS alloys (Figure 3a,b).

### 3.2. Wear Characterization

#### 3.2.1. XPS Analysis

**Figure 4:**
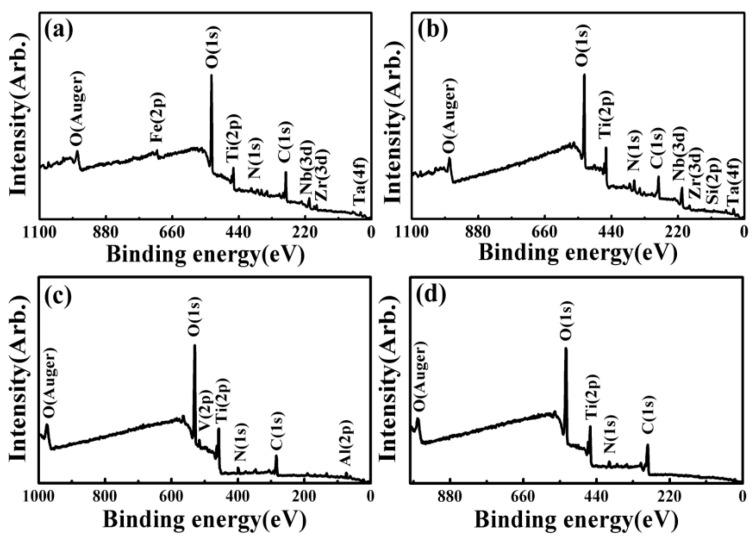
Full range XPS spectra of the worn surfaces after the wear tests under the 6.5 N-1 Hz-5 mm-60 min condition: (**a**) TNZTF; (**b**) TNZTS; (**c**) TC4; (**d**) CP-Ti.

**Figure 5:**
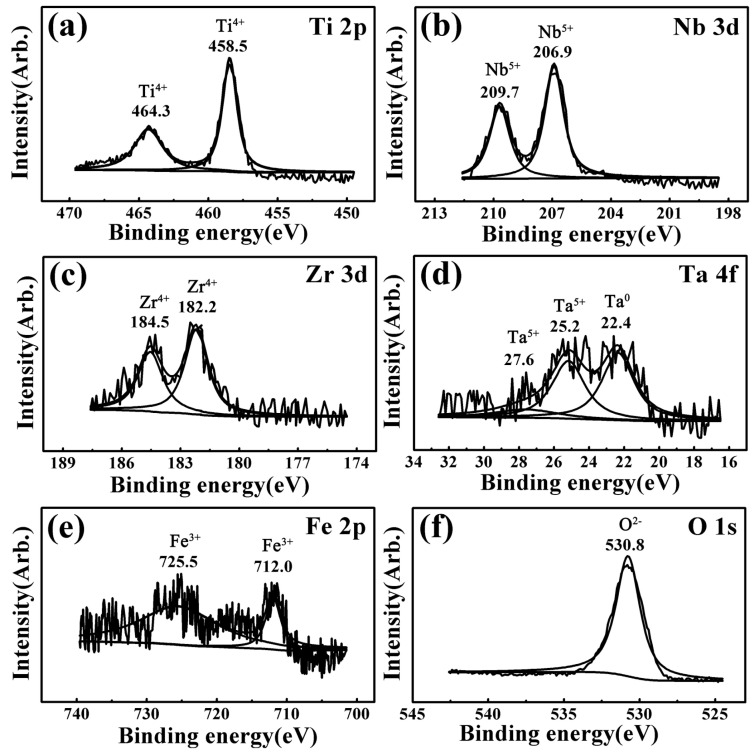
Narrow spectrum scanning XPS spectra of the worn surface for the TNZTF sample after the wear tests under the 6.5 N-1 Hz-5 mm-60 min condition: (**a**) Ti 2p; (**b**) Nb 3d; (**c**) Zr 3d; (**d**) Ta 4f; (**e**) Fe 2p; (**f**) O 1s.

**Figure 6:**
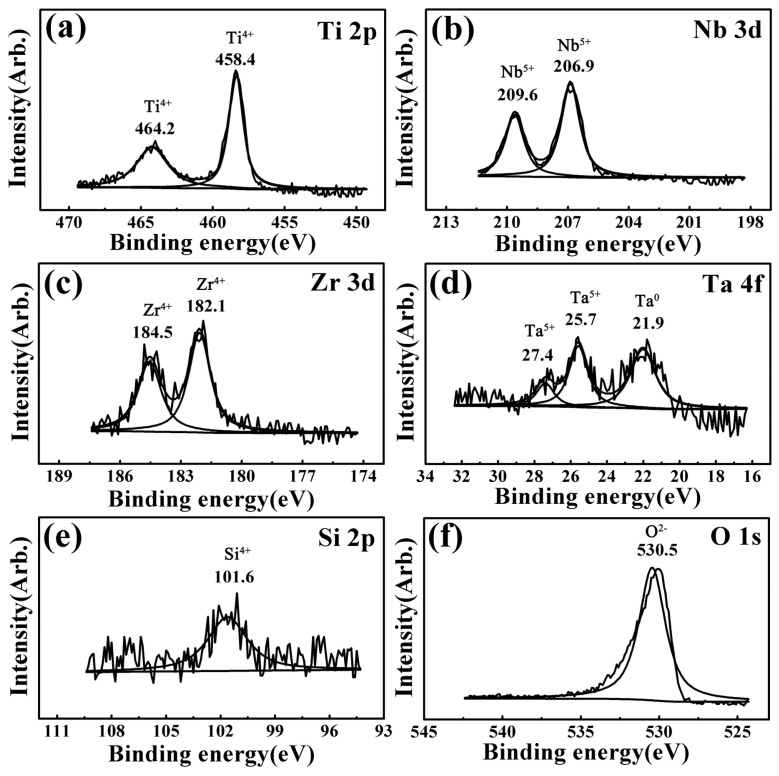
Narrow spectrum scanning XPS spectra of the worn surface for the TNZTS sample after the wear tests under the 6.5 N-1 Hz-5 mm-60 min condition: (**a**) Ti 2p; (**b**) Nb 3d; (**c**) Zr 3d; (**d**) Ta 4f; (**e**) Si 2p; (**f**) O 1s.

**Figure 7:**
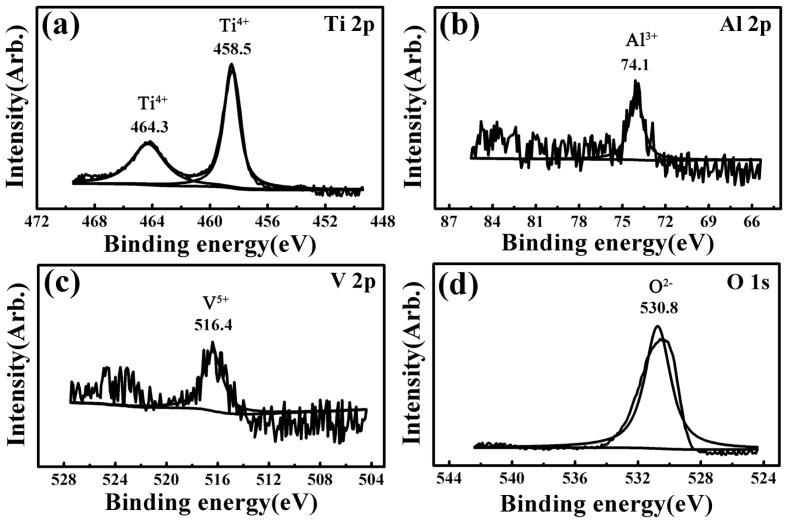
Narrow spectrum scanning XPS spectra of the worn surface for the TC4 sample after the wear tests under the 6.5 N-1 Hz-5 mm-60 min condition: (**a**) Ti 2p; (**b**) Al 2p; (**c**) V 2p; (**d**) O 1s.

**Figure 8:**
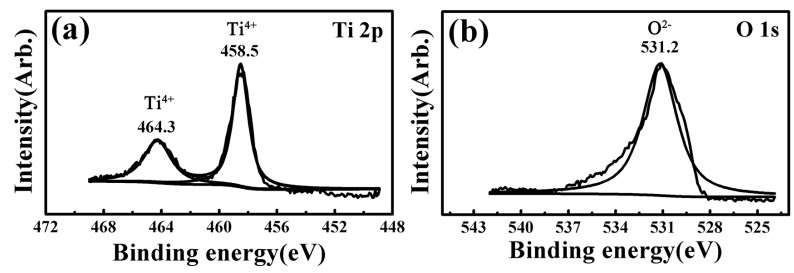
Narrow spectrum scanning XPS spectra of the worn surface for the CP-Ti sample after the wear tests under the 6.5 N-1 Hz-5 mm-60 min condition: (**a**) Ti 2p; (**b**) O 1s.

XPS spectra results of the worn surfaces for all the investigated samples were almost the same under different wear conditions. Taking the 6.5 N-1 Hz-5 mm-60 min wear condition as a representative, Figure 4, Figure 5, Figure 6, Figure 7 and Figure 8 present the full range and narrow spectrum scanning XPS spectra to analyze the chemical composition of the worn surfaces. Table 3 presents the binding energy values and chemical states of the main XPS peaks for the worn surfaces. The spectrum peaks from alloy constituents were composed of peaks of oxidized states and metallic states.

According to the narrow spectrum scanning XPS spectra (Figure 5, Figure 6, Figure 7 and Figure 8) of each sample, the binding energy of the main peaks for each element was obtained. The chemical states of each element were determined according to the XPS database and literature [38,40,43,58,59,60,61,62,63]. For TNZTF (Figure 5), Ti 2p spectra were decomposed into two components at 458.5 and 464.3 eV corresponding to Ti^4+^ oxidation state, which are related to TiO_2_ [38,40,43,58,63]. The same doublets but with a slight shift were found at the Ti 2p spectra of TNZTS (Figure 6), TC4 (Figure 7), and CP-Ti (Figure 8). Nb 3d spectra of TNZTF (Figure 5) were formed by two doublets at 206.9 and 209.7 eV, which corresponded to the oxidation state of Nb^5+^ [38,43,63]. Nb 3d spectra for TNZTS (Figure 6) also contained two doublets for Ti^4+^ (206.9 eV, 209.6 eV) that were related to Nb_2_O_5_. Zr 3d spectra presented two doublets at 182.2 and 184.5 eV for TNZTF (Figure 5), and 182.1 and 184.5 eV for TNZTS (Figure 6), corresponding to the Zr^4+^ oxidation state [38,43,58]. Ta 4f spectra of TNZTF (Figure 5) revealed three contributions at 22.4 eV, 25.2 eV, and 27.6 eV, corresponding to Ta^0^ (metallic state), Ta^5+^ (oxidation state), and Ta^5+^, respectively. The same doublets were found at the Ta 4f spectra of TNZTS (Figure 6), with a slight shift. For Fe 2p spectra of TNZTF (Figure 5), minor peaks observed at 712.0 and 725.5 eV may be related to Fe^3+^ [38]. Minor peak observed at 101.6 eV of Si 2p spectra for TNZTS (Figure 6) may be related to Si^4+^ [63]. For TC4 (Figure 7), Al 2p spectra were formed by one doublet at 74.1 eV, which probably corresponded to the Al^3+^ oxidation state [43], and V 2p spectra had one doublet at 516.4 eV, probably corresponding to the V^5+^ oxidation state. O 1s spectra (Figure 5, Figure 6, Figure 7 and Figure 8) revealed the presence of the O^2−^ signal at 530.8, 530.5, 530.8, and 531.2 eV, associated with the aforementioned metal oxide [38,40,58,63]. As shown in Figure 4, the little amount of C and N detected was probably from the impurities of the investigated samples due to environmental contamination [40].

In brief, the worn surfaces of all the samples showed the presence of TiO_2_. In addition, the worn surfaces of TNZTF and TNZTS also covered Nb_2_O_5_, ZrO_2_, and a small amount of Ta_2_O_5_ and Ta element. Trace Fe_2_O_3_ and SiO_2_ were also detected for the TNZTF and TNZTS alloys, respectively. For the TC4, the worn surface also contained Al_2_O_3_ and V_2_O_5_ in addition to TiO_2_. The worn surface of the CP Ti mainly included TiO_2_.

#### 3.2.2. Wear Volume

**Figure 9:**
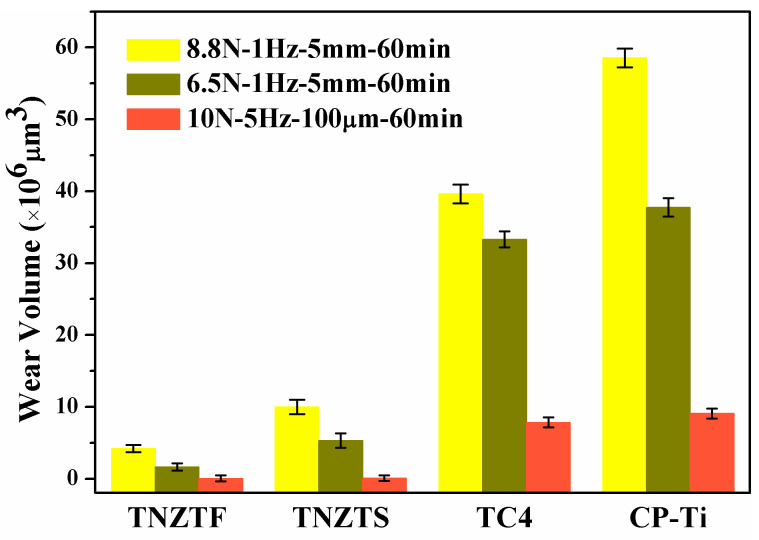
Wear volume of the four samples under sliding and fretting wear conditions.

Figure 9 displays the wear volume of the four materials after wear tests. It is obvious that the wear volume increased as the load increased from 6.5 to 8.8 N under the sliding wear conditions. Furthermore, the wear volume in the sliding wear test was larger than that in the fretting wear test. The variation in the wear volume was TNZTF < TNZTS < TC4 < CP-Ti under the sliding and fretting wear conditions. This indicates that the variation of wear resistance was TNZTF > TNZTS> TC4 > CP-Ti. In general, the anti-wear property was directly in proportion to the hardness of a material [37,39,52,63]. Moreover, the finer grain size also contributed to the increase in hardness according to the Hall–Petch equation [52,64,65]. Wood et al. [32] also reported that nanostructured titanium alloy surfaces experienced considerably lower friction. This probably shows that the higher wear resistance of the fabricated TNZTF and TNZTS alloys benefitted from their higher hardness and finer grain size. The result acquired in this work is consistent with that reported by Pan et al. [52], Zou et al. [64], and Attar et al. [65].

Significantly, the wear volume of the TNZTF and TNZTS samples was far less than that of the TC4 and CP-Ti samples. This outstanding anti-wear ability is mainly attributed to the high hardness of the TNZTF and TNZTS samples. Moreover, hard oxides formed on the surface and the finer grain size both contributed to the increase in surface hardness. Compared with TiO_2_, Nb_2_O_5_ is a harder oxide and possesses better lubricating properties [8,36,66]. Thus, whether there is hard Nb_2_O_5_ on the surface after wear tests is also one of the main factors leading to the hardness difference for the four materials. According to the XPS analysis results, as shown in Figure 4, Figure 5, Figure 6, Figure 7 and Figure 8, the oxides on the surfaces of TNZTF and TNZTS alloys were mainly composed of TiO_2_ and Nb_2_O_5_ after the wear tests. Nevertheless, the oxides on the surfaces of the TC4 and CP-Ti samples were mainly composed of TiO_2_. Furthermore, the fact that the addition of Nb and Zr could enhance wear resistance by forming a protective Nb_2_O_5_ and ZrO_2_ film on the alloy surface has been proven by other researchers [36,38,43,58].

#### 3.2.3. 3D Optical Micrographs of Wear Scars

**Figure 10:**
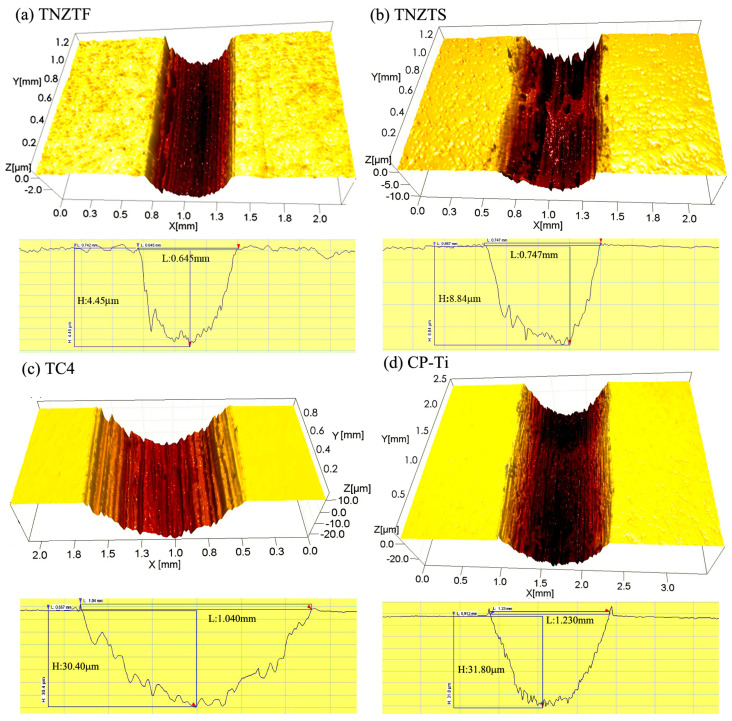
Cross-section view of 3D optical micrographs and the depths and widths of the wear scars from different samples after the wear tests under the 8.8 N-1 Hz-5 mm-60 min condition.

Figure 10 shows a cross-section view of 3D optical micrographs and the depths (H) and widths (L) of wear scars from the four samples after the sliding wear tests under the 8.8N-1Hz-5mm-60min condition. Apparently, the grooves were parallel to the sliding wear direction. This is the main characteristic of the abrasive wear mechanism. The result acquired here is in accordance with that reported by Diomidis et al. [46]. The widths of the wear scars were 0.645 ± 0.005, 0.747 ± 0.005, 1.040 ± 0.013, and 1.230 ± 0.015 mm (Figure 10a–d), and the depths were 4.45 ± 0.04, 8.84 ± 0.05, 30.40 ± 0.15, and 31.80 ± 0.15 μm (Figure 10a–d). As reported by Rajendhran et al. [67], wear width and depth in terms of scratch are the possible means to measure the damage assessment. This has been also reported by other researchers [40,61,68]. The widths and depths of the wear scars for the TNZTF and TNZTS alloys were much smaller under the same sliding wear condition, indicating their higher wear resistance than that of the TC4 and CP-Ti samples. This is mainly attributed to the higher hardness and finer grain size of the fabricated alloys. Moreover, the TNZTS alloy was characterized by slight adhesion wear, owing to its high plasticity (Figure 10b). As shown in Figure 10c,d, the appearance of deep and wide grooves for the TC4 and CP-Ti samples was mainly due to the lower hardness and coarser grain size.

**Figure 11:**
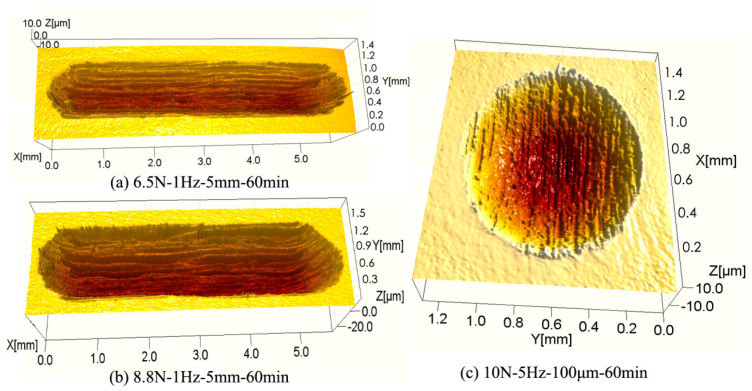
The 3D optical worn surface micrographs of the TC4 sample after the wear tests under different wear conditions.

Figure 11 shows the 3D optical worn surface micrographs of the TC4 sample after sliding and fretting wear tests. The change of worn surface morphology after the wear tests can be observed clearly. Under the sliding wear condition (Figure 11a,b), the width and depth of the wear scar were shallower when the load was 6.5 N (Figure 11a) compared to that of 8.8 N (Figure 11b). Under the fretting wear condition (Figure 11c), the wear scar in the central area was more severe than that in the edge area. Moreover, the wear volume was smaller than that under the sliding wear condition, being consistent with the calculated wear volume (Figure 9).

#### 3.2.4. Comparative Analysis of Wear Morphology and Wear Mechanism

**Figure 12:**
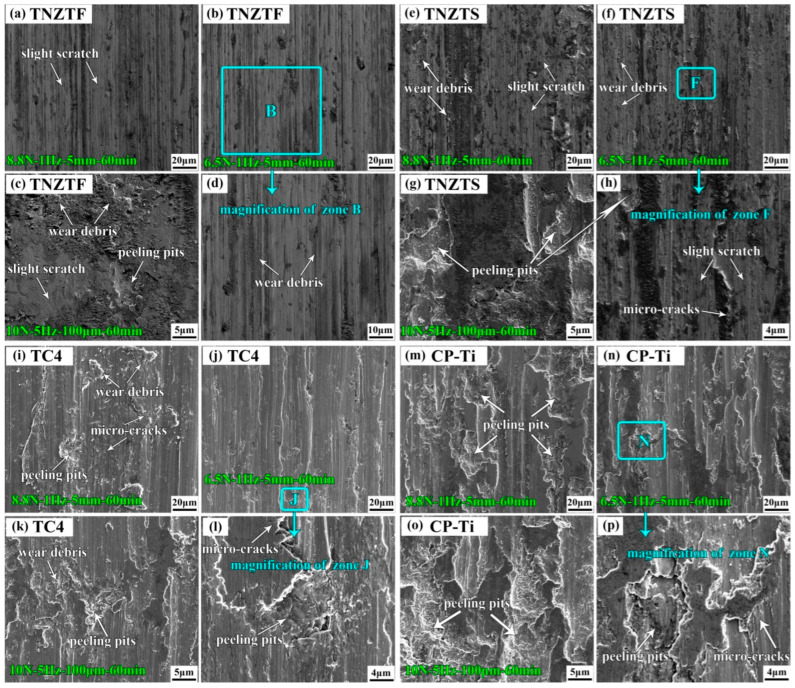
SEM morphologies of the worn surfaces from the four samples after the wear tests under different wear conditions: (**d**) Enlarged view of zone B in (**b**); (**h**) Enlarged view of zone F in (**f**); (**l**) Enlarged view of zone J in (**j**); (**p**) Enlarged view of zone N in (**n**).

Figure 12 shows SEM morphologies of the worn surfaces from different samples after the wear tests. By comparing all the SEM morphologies (Figure 12a–p), it is interesting to find out that some black materials in the shape of fish scales can be seen clearly from the TNZTF alloy (Figure 12a–d) and TNZTS alloy (Figure 12e–h), being the oxide layer generated during the wear tests. The oxide layer could play a protective role for the matrix material. However, there was no such obvious phenomenon for the TC4 (Figure 12i–l) and CP-Ti (Figure 12m–p) samples.

Under the sliding wear condition of 6.5 N-1 Hz-5 mm-60 min (Figure 12b,d,f,h,j,l,n,p), since the relative motion displacement of the two contact surfaces was much larger than their contact radius, uniform plastic deformation and grooves along the sliding direction appeared on the four sample surfaces. Compared with the TNZTF alloy (Figure 12b), the grooves of the TNZTS alloy (Figure 12f) were not as obvious, which was because the material that squeezed into the side of the grooves was quickly flattened out, owing to its relatively lower hardness. Wear debris (Figure 12b,d,f,h) appeared on the surface under the action of repeated deformation and low cycle fatigue. A few micro-cracks (Figure 12h) appeared for the TNZTS alloy, which may have been fatigue cracks [26,69]. The slight scratch (Figure 12b,d,f,h) showed a feature of the abrasive wear mechanism. In addition, a few peeling pits (Figure 12h) formed on the surface of the local area due to the high plasticity of the TNZTS alloy, which is characteristic of slight adhesive wear. Nevertheless, for the TC4 alloy (Figure 12j,l), obvious plastic deformation and grooves along the sliding direction appeared on the surface (Figure 12j). The metal in the groove moved to both sides and then both sides were uplifted, which is a typical feature of abrasive wear. Relatively more micro-cracks (Figure 12l) appeared on the surface, showing characteristics of fatigue wear. Moreover, peeling pits (Figure 12l) on the surface showed the characteristics of adhesive wear. For the CP-Ti sample (Figure 12n,p), even if the loading force was relatively low, a large area of plastic deformation layer peeled off, showing the characteristics of adhesive wear. Plastic deformation and plows indicated that there were characteristics of abrasive wear. Micro-cracks (Figure 12p) appeared on the surface, clearly showing a feature of fatigue wear. The TNZTF and TNZTS alloys exhibited much more smooth surface morphology than the TC4 and CP-Ti samples, indicating their higher anti-wear properties.

Under the sliding wear condition of 8.8 N-1 Hz-5 mm-60 min (Figure 12a,e,i,m), as the load increased to 8.8 N, the wear degree of the four materials increased significantly compared with that under the load of 6.5 N (Figure 12b,f,j,n). For the TNZTF and TNZTS alloys shown in Figure 12a,e, the grooves were deeper, and more wear debris formed on the surface. Meanwhile, the wear scars appeared as slight scratches, indicating the characteristic of an abrasive wear mechanism. Moreover, a few micro-cracks (Figure 12e) appeared for the TNZTS alloy that may be fatigue cracks. For the TC4 alloy (Figure 12i), deeper grooves made the surface rough. The contact stress exceeded the yield strength of the material, leading to severe plastic deformation. Part of the transferred metals directly fell off, and the peeling phenomenon of local material was more serious, indicating that the adhesive wear was aggravated. Meanwhile, more micro-cracks (Figure 12i) can be seen, indicating a typical feature of fatigue wear. Silva et al. [40] also reported grooves and micro-cracks on the TC4 surface after wear. This demonstrates that the wear mechanism of the TC4 alloy is the joint action of adhesive wear and fatigue wear under this condition. A more serious peeling phenomenon (Figure 12m) illustrates that the wear mechanism is mainly adhesive wear for the CP-Ti sample. The TNZTF and TNZTS alloys still exhibited more smooth surface morphology than that of the TC4 and CP-Ti samples, indicating their higher wear resistance. The narrower and shallower grooves of the TNZTF and TNZTS alloys were mainly attributed to their finer grain size and higher hardness. Remarkably, there was almost no peeling phenomenon for the TNZTF alloy, owing to its higher hardness.

Under the fretting wear condition of 10 N-5 Hz-100 μm-60 min (Figure 12c,g,k,o), the relative motion displacement of the two contact surfaces was smaller than their contact radius. Thus, for the TNZTF alloy (Figure 12c), plastic strain was concentrated in the local area, and large amounts of wear debris are difficult to excrete. The presence of a few peeling pits indicated the characteristics of a slight adhesive wear mechanism. Wear scars appeared as slight scratches, showing the abrasive wear mechanism. As for the TNZTS alloy (Figure 12g), grooves were concentrated in the local area, indicating the characteristic of the abrasive wear mechanism. More peeling pits appeared on the surface. Moreover, the local peeling area was larger and rougher than that of the TNZTF alloy, which was the joint action of the adhesive wear and peeling wear. For the TC4 alloy (Figure 12k), a large amount of wear debris and peeling pits appeared on the surface. Along the fretting wear direction, a large area of delamination occurred in addition to obvious furrows. This displays characteristics of joint action of abrasive wear mechanism and adhesive wear mechanism. As for the CP-Ti sample (Figure 12o), a large area of peeling pits and obvious grooves appeared on the surface, demonstrating that the wear mechanism was a joint action of severe adhesive wear and abrasive wear. A similar mechanism was described also by other researchers [38]. In brief, relatively less peeling pits appeared for the TNZTF and TNZTS alloys, suggesting higher wear resistance than the TC4 and CP-Ti samples. The four materials all exhibited more rough surface morphology than that under sliding wear conditions because of the higher load and frequency under fretting wear conditions.

## 4. Conclusions

In this work, the ultrafine-grained TNZTF and TNZTS alloys fabricated by mechanical alloying and spark plasma sintering exhibited much better wear resistance compared with TC4 and CP Ti under the sliding and fretting wear tests in Hank’s balanced salt solution at a body temperature of about 37 ± 1 °C. The excellent wear resistance can mainly be attributed to the higher hardness and ultrafine-grained microstructure of the fabricated alloys. The addition of Nb and Zr further improved the wear resistance by forming a protective Nb_2_O_5_ and ZrO_2_ oxide film. The comparatively discussed wear mechanisms further confirm the reason for the excellent wear resistance. Under the sliding wear tests, the wear mechanisms of the fabricated TNZTF and TNZTS alloys were mainly characterized by abrasive wear, while adhesive wear, abrasive wear, and fatigue wear were found for TC4 and CP Ti. Under the fretting wear tests, all the investigated samples were dominated by different degrees of adhesive wear mechanism and abrasive wear mechanism, and they exhibited more rough surface morphologies than those under sliding wear conditions because of the higher load and frequency. The outstanding anti-wear ability of the fabricated titanium alloys could be of great significance for biomedical alloy development and implant design.

## Figures and Tables

**Table 1:** Mechanical properties of the investigated alloys. TNZTF: Ti_65_Nb_23.33_Zr_5_Ta_1.67_Fe_5_; TNZTS: Ti_65_Nb_23.33_Zr_5_Ta_1.67_Si_5_; TC4: Ti-6Al-4V; *σ*_0.2_: yield stress; *σ*_max_: ultimate stress; *ε*_f_: fracture strain.

Samples	Phase Constituents	*σ*_0.2_ (MPa)	*σ*_max_ (MPa)	*ε*_f_ (%)	Hardness (HV)
TNZTF	β-Ti + FeTi_2_	2247 ± 5	2872 ± 5	23 ± 1	483 ± 4
TNZTS	β-Ti + S2	1347 ± 5	3267 ± 10	58 ± 1	378 ± 3
TC4	β-Ti + α-Ti	1005 ± 3	2350 ± 4	43 ± 1	294 ± 4
CP-Ti	α-Ti	402 ± 2	1151 ± 3	73 ± 2	180 ± 3

**Table 2:** The composition of the Hank’s balanced salt solution.

Components (Inorganic Salts)	Molecular Weight	Concentration (mg/L)
CaCl_2_	111.0	140
MgCl_2_·6H_2_O	203.0	100
MgSO_4_·7H_2_O	246.0	100
KCl	75.0	400
KH_2_PO_4_	136.0	60
NaHCO_3_	84.0	350
NaCl	58.0	8000
Na_2_HPO_4_	142.0	48
Glucose	180.0	1000

**Table 3:** Binding energy values and chemical states of XPS results of the worn surfaces.

Samples	Ti 2p	Nb 3d	Zr 3d	Ta 4f	Fe 2p	Si 2p	Al 2p	V 2p	O 1s
TNZTF	458.5	206.9	182.2	25.2	712.0	-	-	-	530.8
	464.3	209.7	184.5	27.6	725.5				-
TNZTS	458.4	206.9	182.1	25.7	-	101.6	-	-	530.5
	464.2	209.6	184.5	27.4		-			-
TC4	458.5	-	-	-	-	-	74.1	516.4	530.8
	464.3						-	-	-
CP-Ti	458.5	-	-	-	-	-	-	-	531.2
	464.3								-

## Data Availability

Data are contained within the article.
